# Differential expression of proteins in monozygotic twins with discordance of infantile esotropic phenotypes

**Published:** 2011-06-17

**Authors:** Guixiang Liu, Haiqing Bai, Zhiyong Yan, Yuna Ma, Hui Li

**Affiliations:** 1 Department of Ophthalmology, the Affiliated Hospital of Medical College, Qingdao University, Qingdao, China; 2 Department of Microbiology, Medical College, Qingdao University, Qingdao, China

## Abstract

**Purpose:**

To identify strabismus-related proteins, we performed proteome analysis in monozygotic twins with discordance of congenital esotropic phenotypes and in normal children.

**Methods:**

Surface-enhanced laser desorption/ ionization time-of-flight mass spectrometry (SELDI-TOF-MS) technology was used to detect changes in protein expression in a pair of twins with discordant esotropic phenotypes (twin A is orthotropic and twin B is esotropic). In addition, two non-twin esotropic children and two orthotropic children of the same age were chosen. The differentially expressed proteome obtained was validated in twelve non-twin esotropic children and eighteen orthotropic children and compared to the protein database.

**Results:**

We detected four differentially expressed proteins in monozygotic twins with discordance of congenital esotropic phenotypes. The corresponding molecular weights were 4,146 Da, 4,801 Da, 7,786 Da, and 5,859 Da, respectively. Among these 4 proteins, the first three proteins were down-regulated and the last was upregulated. The initial characterization of these detected proteins via protein library revealed that their characteristics were similar to those of the glucagon precursor, pituitary adenylate cyclase-activating polypeptide (PACAP), camp-dependent protein kinase inhibitor α, and anti-metastasis gene (antigen), respectively.

**Conclusions:**

There were differentially expressed proteins between monozygotic twins with discordance of congenital esotropic phenotypes and normal children. These differentially expressed proteins were mainly down-regulated in the strabismus patients and may be involved in the occurrence and development of congenital esotropia.

## Introduction

To date, the cause of infantile esotropia is unknown. Previous studies found that patients with strabismus usually had positive family history and the incidence of the strabismus is much higher in monozygotic twins than in multizygotic twins, indicating that genes may play a role in infantile esotropia [1-3]. However, it was also found that there were large individual differences in the strabismus phenotype in monozygotic twins although DNA sequences and growth environments are identical in those patients. This indicates that other factors may also be related to the development of strabismus. Therefore, it is important to study the related factors to better understand the etiology of strabismus. In recent years, the development of proteomics and gene chips has provided new avenues to study the etiology of strabismus. In this study, using a technique called surface-enhanced laser desorption/ionization time as determined by time-of-flight mass spectrometry (SELDI-TOF-MS), we analyzed serum protein spectra and screened out differentially expressed proteins in a pair of monozygotic twins with discordance infantile esotropia and in children with/without infantile esotropia.

## Methods

### Study subjects; selection of monozygotic twins

The monozygotic twins with discordance of strabismic phenotypes was defined as a pair of twins in which one member had one type of strabismus, while the other was orthotopic or had a different type of strabismus [1]. The inclusion criteria were as follows: A) twins that were of the same gender; B) determination by obstetricians at birth; and C) similarity in phenotype, as determined by dermatoglyphics, hair color, eye color, nose shape, face shape, lip shape, eyelid shape, dentition, and other aspects of phenotype. Finally [4], using 10 pairs of fluorescent-labeled short tandem repeat (STR) primers with a high degree of heterozygosity in the Chinese population and STR as a gene marker, we performed genotyping analysis. Based on the concordance of phenotypes, we conducted zygosity identification.

### Experimental design

#### Selection of protein markers

The experimental group included twin set B and two non-twin congenital esotropia patients who were similar in age, gender, bodyweight, and other general characteristics to twin set B. The control group included twin set A and two healthy children who were similar in appearance to twin set A but without strabismus, other related eye diseases, or systemic disease.

#### Validation of protein markers

Twelve children with infantile esotropia and eighteen healthy children were chosen to validate the differentially expressed proteins. This information revealed that ages and gender were fairly well distributed among the experimental group and the control group.

### Instruments and reagents

PBSII C-type surface-enhanced laser desorption/ionization time-of-flight mass spectrometry (SELDI-TOF-MS) and its software Protein Chip Software3.0, energy-absorbing molecular CHCA and the H4-type protein chip were obtained from Ciphergen Ciphergen biosystem Inc. (Fremont, CA). HEPES, CHAPS, HPLC-grade ultra-pure water and other reagents were from Sigma-Aldrich (St. Louis, MO).

### Procedures

#### Collection and pre-processing of serum

We collected 4–5 ml of whole blood from each subject and put the blood into clean test tubes, then the tubes were immediately placed at 4 °C for 4 h. The blood samples were then centrifuged at 990× g for 20 min to remove hemolytic specimens, which were further centrifuged at 1,760× g for 5 min to remove residual cell debris and to obtain the serum. The serum was poured into another new 100 μl centrifuge tube and stored at −80 °C for future use. Before each experiment, the serum sample was placed on ice to melt, and it was then centrifuged at 15,860× g for 10 min to remove the insoluble material. Next, 10 μl of serum was taken and mixed with 20 μl U9 buffer (9 mol/l urea, 20 g/l CHAPS, 50 mmol/l Tris-HCI, 10 g/l DTT, pH 9.0) and shaken in an ice bath for 30 min at 300 r/min. We then added 360 μl WCX-2 buffer (50 mmol/l NaAc, pH4.0), and the solution was placed on ice and quickly mixed.

#### Pretreatment with WCX-2 chips

The chips were loaded into a biochip processor and 200μl WCX-2 buffer was added to each hole. The processor was placed in the oscillator for 5 min at 300 rpm, and the buffer was then discarded and the operation repeated once again.

#### Sample testing

We added 100 μl serum to each hole and then shook the samples for 1 h at 300 rpm in the oscillator. Then samples were thrown off and washed with 200 μl WCX-2 buffer two times at 300 r/min in the oscillator at room temperature. Next, 200μl HEPES (1 mmol/l, pH4.0) was added in and immediately shaken off. We opened the chip processor, took out the chips, dried them, and then added 0.5 μl of sinapinic acid (SPA) onto each point. After drying, we again added SPA and held for further testing.

#### TOF data acquisition for serum proteins

A PBSII C-type mass analyzer and Ciphergen protein chip 3.1 software were used to read and collect the data. Prior to the data collection, the instrument was calibrated by the All-in-one standard protein NP20 chip calibration instrument, the error was kept within 0.1%. The instrument was set to automatically collect and process the data: the laser intensity was set to 210; the detection sensitivity was set at 9; the optimized molecular weight range was 2–10 Da; the highest molecular weight was 50,000 Da. One hundred-thirty collections were performed at each point and different locations.

#### Screening of differentially expressed proteins

Twin set B and two other patients with congenital esotropia were grouped as the experimental group; twin set A and two other healthy children were grouped as the control group. We wanted to determine whether the proteins were differentially expressed between the groups. First, we removed background noise (baseline) of the protein spectra from the TOF data acquired from these two groups, and then normalize with the total ion current and the peak serum protein of 6,638 Da, which exists stably in human sera, to reduce experimental error. At last, the Biomarker Wizard software was then used to calculate the difference of protein peak (p value) among groups and to identify those differentially expressed proteins.

#### Validation of differentially expressed proteins

The screening of differentially expressed proteins mentioned above were verified and analyzed with serum protein spectra from twelve children with congenital esotropia and eighteen healthy children using Biomarker Wizard software.

## Results

### Selection of monozygotic twins

The probability that the selected twins will be monozygotic is 99.95%. These twins were 2-year-old males, born at 32 weeks. The bodyweights at birth for twin set A and B were 2,300 g and 2,200 g, respectively. The parents reported that twin set B became esotropic at 1 month old. The twins were normal in refraction and anterior and posterior segment examination. In twin set B, the left eye was obviously esotropia, ocular motor examination showed an overaction of the left inferior oblique muscle. The diagnosis for twin set B was infantile esotropia. Eye position and eye movement were normal for twin set A. Their parents were orthotropia and without other eye diseases.

### Screening of differentially expressed proteins

**Figure 1:**
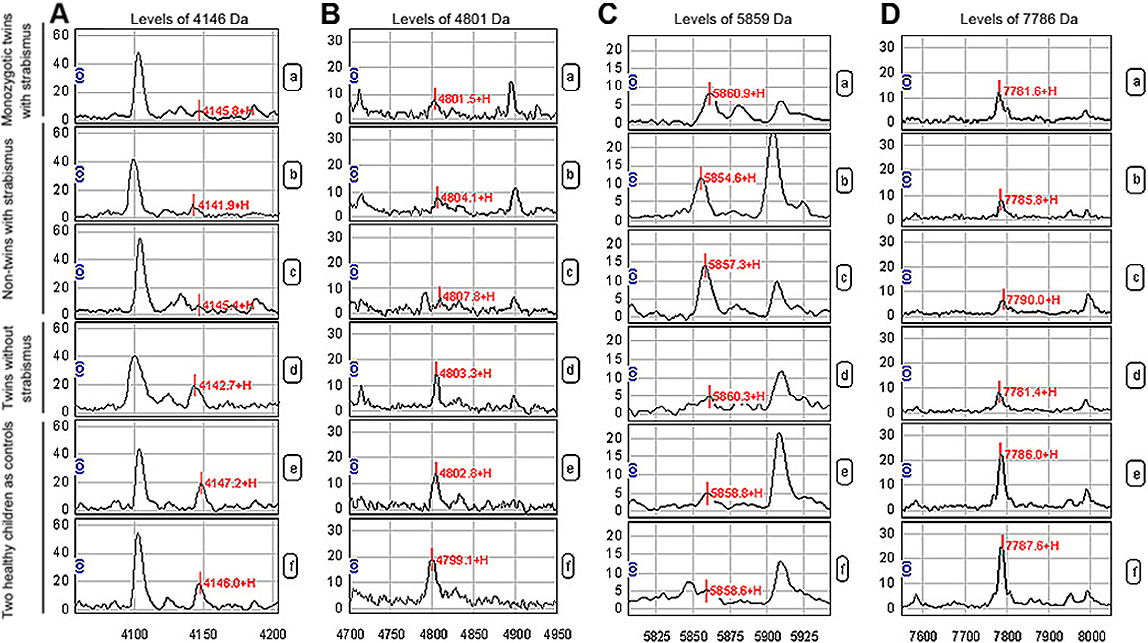
Proteins that are differentially expressed between children with and without strabismus. Abscissa is m/z (Da), the axis is the relative area of the protein peak (S/N). a. monozygotic twins with strabismus; b and c, non-twins with strabismus; d, twins without strabismus; e and f, two healthy children as controls. Levels of 4,146 Da protein (**A**) and 4,801 Da protein (**B**) were lower in children with strabismus (a, b, and c), as compared to non-strabismus children (d, e, and f); Protein 5,859 Da (**C**) was highly expressed in strabismus children (a, b, and c), as compared to non-strabismus children (d, e, and f). Protein 7,786 Da (**D**) was expressed at lower levels in twins (a and d) and strabismus children (b and c), as compared to healthy controls (e and f).

We analyzed the protein spectra from the experimental group and the control group using Biomarker Wizard software and screened out four differentially expressed protein peaks. Among these, the molecular weight of the 4,146 Da and 4,801 Da protein peaks were lower in the sera including twin set B and two non-twin infantile esotropia patients as compared to healthy children (Figure 1A,B); the molecular weight of the 5,859 Da protein peak was higher in the sera of patients than in the healthy children (Figure 1C); and the molecular weight of the 7,786 Da protein peak was lower in sera of patients and twin set A than in the healthy children (Figure 1D).

### Verification of differentially expressed proteins

The four differentially expressed proteins were verified using serum protein spectra from 30 cases including twelve children with infantile esotropia and eighteen healthy children. The results are shown in Table 1.

**Table 1:** Comparison of four proteins that are differentially expressed in the congenital esotropia and control groups (Means±S).

**Peak mass**		**Experimental group**		**Control group**	
**n**	**M/Z**	**n**	**Intensity**	**n**	**Intensity**
30	4146.0±2.28	12	7.29±0.33	18	18.92±0.97^a^
30	4801.3±1.56	12	7.56±0.84	18	16.89±1.21^b^
30	5859.4±3.13	12	11.35±2.14	18	5.00±0.78^c^
30	7786.7±4.32	12	8.84±1.55	18	23.64±1.81^d^

Compared with the control group, ^a^t’=46.96, p<0.01; ^b^t=23.18, p<0.01; ^c^t’=9.85, p<0.01; ^d^t=23.19, p<0.01.

In Table 1, peak mass is represented as the molecular weight of the protein peak. In spite of there were slight differences in the molecular weight of the same protein peak in different TOF series, the protein peaks of coming from different samples but in the tolerance interval of molecular weight of protein were considered to represent the same protein, which the protein peaks from 30 samples was used to the masses of normalize with Biomarker Wizard software, and to calculate the average and standard errors for each protein peak. The term “m/z” denotes the mass-to-charge ratio of the protein peak. Because of each protein peak carried one charge, m/z=m, where “m” denotes the molecular weight represented by the protein peak. “Intensity” means the ratio of intensity/contents of the protein peak, which derived by subtracting baseline values from the protein peak S/N and then normalizing by the “total ion” value.

### Preliminary prediction of differentially expressed proteins

We searched the ExPASy protein library (Expert Protein Analysis System) using the initial features of these differentially expressed proteins, including molecular weights (MW), isoelectric (pI, according to the pH value of the buffer used to treat samples), organism and location (serum) to identify database proteins that matched our detected proteins. We found that proteins of 4,146 Da, 4,801 Da, 5,859 Da, and 7,786 Da were similar to glucagon precursor, pituitary adenylate cyclase-activating polypeptide precursor (PACAP), metastasis-suppressor KiSS-1 precursor (Kisspeptin-1) and cAMP-dependent protein kinase inhibitor alpha, respectively.

## Discussion

Currently, most researchers think that infantile esotropia is a multi-factorial disease. The scholars who support nerve-factor theory, which is denoted the “Outgoing Principle,” believe that information on eye position provided by the CNS is transmitted to extraocular muscles by efferent nerves. Any abnormality in this pathway will cause abnormalities of eye position [5]. Therefore, we sought to determine whether there was any abnormality in the transmission of signals from the CNS to the extraocular muscle that was discernible through the study of proteomics. The proteome comprises all proteins encoded by the individual’s genome. Inter-individual comparisons of proteomes under normal or disease conditions may reveal a specific type or group of proteins related to the disease, provide the best combination of indicators for disease diagnosis and a theoretical basis and new approach to disease control and prevention, as well as clarify the underlying mechanism. By combining mass spectrometry and chromatography and other technical principles, SESELDI-TOFMS allows large-scale testing of original samples such as sera, urine, etc. This technique can dissociate all types of proteins for flight mass spectrometry based on flight time in the field. The information obtained is presented as peaks that are intuitive. With SESELDI-TOFMS, one can rapidly acquire proteome-related information. The benefits of this technique include a high resolution, a high level of reproducibility and ease of operation. The approach is widely applied in the field of proteome study and has been successful in identifying specific protein markers in diseases, such as ovarian, bladder, breast and prostate cancer [6-8].

Because of an individual’s proteome is influenced by the living environment, dining habits, age, gender and other factors, there is great variability in protein expression, so, proteome studies of common cases must investigate large sample to exclude the interference of non-specific causal factors. This greatly increases the cost of the research. Monozygotic twins have identical genetic backgrounds and higher-than-average levels of consistency in their proteome. Therefore, the interference of non-specific proteins to the study of differential expression proteins is greatly reduced. Consequently, it is relatively easy to identify disease-specific proteins in monozygotic twins. Such twins are the ideal subjects for proteome analyses that aim to identify differentially expressed proteins. In tuis study, follow-up surveys confirmed that the monozygotic twins have been living in the same environment since birth, further reducing the interference from non-specific proteins. At first, we performed flight mass spectrometry in the set of twins, as well as a few esotropic children and healthy children that were matched with the twins studied, and do small sample compare so as to identify differentially expressed proteins, and then verify the 4 differentially expressed proteins in a large sample. This strategy allowed us to exclude the interference from different genetic backgrounds and living environments. As a result, we did not need to study two large populations, which simplified the experiments and reduced costs. Our results will be important to elucidate the etiology and genetics of this disease.

The twins in this study were identified as monozygotic twins. Twin set B appeared to suffer from congenital esotropia, while twin set A appeared to be normal. These twins have identical genetic backgrounds, so the phenotype difference between individuals should be caused by acquired, rather than genetic, factors. The molecular mechanism underlying this phenotype difference should involve epigenetic changes. In contrast to classical genetic mechanisms, which supply the templates necessary to synthesize proteins, epigenetics controls when, where, and how to use the genetic message.

Throughout life, epigenetic mechanisms allow individuals to respond and constant adapt to environment information [9]. Notably, epigenetic phenomena not only can be inherited independently but also there are complicated interrelationships among them. Sometimes disturbances in epigenetic mechanisms will lead to abnormalities in gene expression or gene silencing, which can result in dysfunction or disease. Previous reports on the cause of discordance of strabismic phenotypes in monozygotic twins also highlight the role played by intrauterine environmental factors. Monozygotic twins share one placenta, with vascular anastomosis between arteries and veins in some placentas. In such situations, blood circulation through the vascular anastomosis results in twin transfusion syndrome due to an imbalanced placental vascular blood flow. The blood recovered by the umbilical artery of one twin enters the umbilical artery of the other twin via this anastomosis to supply nutrients. This blood lacks oxygen, which may lead to the differences in development that can result in disease in one twin [10,11]. Thus, differences in the intrauterine blood and oxygen supply may cause the imbalance of eye development and result in a discordance of strabismic phenotypes. However, the molecular mechanism underlying the phenotype discordance caused by variation in gene expression and environmental factors remains unknown.

Fraga and coworkers [12] found there were obviously differences in DNA methylation level and histone site idio-acetylation between twins by studying discordant phenotypes of 40 sets monozygotic twins. Our results showed that esotropic children express lower levels of 4,146 Da, 4,801 Da, and 7,786 Da proteins but high levels of a 5,859 Da protein. By searching the protein database, we found that the three proteins with reduced expression were similar to glucagon precursor, pituitary adenylate cyclase-activating polypeptide (PACAP), and cAMP-dependent protein kinase inhibitor α. The highly expressed protein was similar to the anti-metastasis gene (antigen). Glucagon precursor not only regulates the absorption of nutrients and the stability of the internal environment at multiple levels but also regulates nervous system development by acting as a central nervous system neurotransmitter [13,14]. Recent research also verified that the glucagon precursor is involved in visually guided eye growth regulation [15-17]. If we can confirm that the 4,146 Da protein is indeed the glucagon precursor in our future research, we will be able to verify that the glucagon precursor plays an important role in the occurrence of congenital esotropia. PACAP is a neuropeptide involved in cell metabolism, cellular differentiation and cell proliferation and plays important roles in neuron growth and damage repair. The protein is even regarded as a cerebral protection factor [18].

However, until now, no report has investigated the protein’s relationship to eye neurons and strabismus. We infer that it may be involved in the occurrence and development of strabismus by affecting the growth and development of the nervous system. cAMP-dependent protein kinases act in all types of tissues as second messengers. Type II exists mainly in neurons and regulates axonal regeneration after CNS injury. Under the proper conditions, increased levels of cAMP in neurons can promote axonal regeneration in the injured CNS [19-21]. The proteins that we found to have reduced expression are all related to the development of nerves and or muscles. We think these proteins probably be associated proteins to development of ocular motor nerves and muscles. During the nerves and muscles, certain factors render the intrauterine environment different for each of these twins, mistakes in the course of gene expression or modification into biologically active proteins were happened in twin set B. These mistakes resulted in an abnormal development of nerves or muscles related to eye movement, which affected the CNS pathway responsible for sending out information regarding eye position to the extraocular muscle, ultimately resulting in congenital esotropia. Our additional finding that cAMP-dependent protein kinase expression was also reduced in twins without strabismus indicates that the expression of this protein has family or genetic characteristics.

Currently, our group is expanding the number of cases to further this study, and we plan to isolate the above differentially expressed proteins and confirm their identity using MS-MS and other techniques. Through studies of proteome differences, we will be able to identify protein markers, which can be used in the early diagnosis of strabismus and in the analysis of the disease’s causality and development, and to provide a new way to the study of etiology and pathogenesis of strabismus.
